# The ‘officer effect’ in risk assessment for domestic abuse: Findings from a mixed methods study in England and Wales

**DOI:** 10.1177/14773708231156331

**Published:** 2023-03-07

**Authors:** Andy Myhill, Katrin Hohl, Kelly Johnson

**Affiliations:** College of Policing, London, UK; Department of Sociology and Criminology, University of London, London, UK; 3057 Department of Sociology, Durham University, Durham, UK

**Keywords:** Domestic abuse, coercive control, risk assessment, police

## Abstract

Research on risk assessment for domestic abuse has focused primarily on the predictive validity of specific tools; less attention has been paid to implementation of risk tools by practitioners. This paper presents findings from a mixed methods study in England and Wales. Multi-level modelling reveals an ‘officer effect’ whereby victims’ responses to the Domestic Abuse, Stalking and Harassment and Honour-Based Violence (DASH) risk assessment are influenced by the specific officer that completes the assessment. Specifically, this officer effect is strongest in relation to questions intended to capture elements of controlling and coercive behaviour, and least apparent in relation to identifying physical injuries. We further present findings from field observations and interviews with first response officers that corroborate and help explain the officer effect. We discuss implications for the design of primary risk assessments, victim safeguarding, and the use of police data for predictive modelling.

## Introduction

Risk assessment in policing emerged during the 1990s as a field of research and practice in response to the risk paradigm recognised as helping to structure elements of policework (Ericson and Haggerty, 1997). The literature on risk assessment for domestic abuse (DA) has developed rapidly in recent years. Numerous risk assessment instruments have been developed and, to varying degrees, tested. While some tools, such as the Ontario Domestic Assault Risk Assessment (ODARA, see Hilton et al., 2004), are based on actuarial principles and numerical scores and thresholds, others, such as the Spousal Assault Risk Assessment (SARA, see Kropp and Hart, 2000), seek to structure practitioners’ professional judgement. Further instruments, such as the Brief Spousal Assault Form for the Evaluation of Risk (B-SAFER, Kropp and Hart, 2004) and the Lethality Screen (Messing et al., 2017) have been developed to be used specifically by frontline practitioners as primary risk assessments or risk identification tools (the ODARA also can be used by first responders). Despite this reliance on practitioner administration and professional judgement, the literature to date has focused primarily on specific instruments’ predictive validity in terms of forecasting repeat victimisation. There has been relatively little focus on the usability of instruments, or how they are implemented by practitioners in the field (though see Barlow and Walklate, 2021).

Numerous research studies highlighted inconsistency historically in police response to domestic-related calls for service (see e.g. Kelly, 1999). Police forces in England and Wales began to develop risk models for DA in the early 2000s, in part with a view to structuring officers’ interactions with victims in order to generate ‘a consistent level of information’ to aid and improve investigation and safeguarding (Robinson, 2010:126). In 2009, chief officers endorsed a standard model for identifying, assessing and managing risk: the Domestic Abuse, Stalking and Harassment and Honour-Based Violence risk identification, assessment and management model (DASH). The DASH model is based on a 27 question risk identification interview undertaken by first response officers with victims, and is used currently by the vast majority of forces in England and Wales, as well as by Police Scotland. The importance of the initial DASH interview cannot be overstated: responses to the questions and the level of risk assigned by first response officers in their primary assessment can dictate the subsequent police response, including whether or not the case is reviewed and/or assigned to a specialist unit, the type and level of safeguarding allocated to the victim, and whether the case is referred to partner agencies and support services.

The authors are aware of conscientious officers completing risk assessments with victims of domestic abuse in a thorough and sympathetic way, as well as examples of poor practice. Inconsistent practice has also been documented by Her Majesty's Inspectorate of Constabulary (2014). This situation warrants consideration of the tools and processes intended to support officers in ensuring consistency in the standard of risk assessment and victim engagement. The present study thus speaks to the gap in the literature around implementation of risk assessment tools by frontline police practitioners. The study seeks first to quantify the extent to which police officer characteristics shape victims’ responses to the DASH, and to assess whether this kind of systematic measurement error afflicts some DASH questions more than others. Second, ethnographic and qualitative data is used to corroborate and explain the quantitative findings through examination of the implementation of the DASH risk assessment in practice by police first response officers. We then discuss the implications of an ‘officer effect’ for the use of the DASH and similar risk assessment tools in frontline policing and make recommendations for how this type of measurement error might be reduced.

## The (contested) concepts of risk and coercive control

‘Risk’ is a contested concept, though Mythen (2014) suggests common underlying elements including (situations of) danger that may result in harm, uncertainty as to whether harm will be realised, and ‘futurity’ (risks are things that may happen at some point over time). In respect of the latter, assessing risk can also be regarded as the attempt to predict future outcomes based on data concerning past events.

Mythen (2014:16) suggests risk is a ‘double-edged sword’ as a means of understanding and governing society – while heightened awareness of risk may enable sensible avoidance strategies, it may equally result in harmful risk-aversion. Some observers have recognised a process of ‘risk creep’ (Walklate and Mythen, 2011:100) whereby the concept of risk has been applied to situations where it is not appropriate or helpful. In addition, critics have suggested the reinterpretation of existing activities as discrete risk factors creates a disconnect with wider contexts and structures (see Mythen, 2014). Thus, Barlow and Walklate (2021:899) suggest focusing on ‘a set number of measurable, individualizing factors’ risks ignoring wider structural issues such as gendered social relationships.

In relation to DA specifically, perhaps the biggest area of contention has been whether the primary purpose of risk assessment is in fact prediction, or prevention (see Bennett Cattaneo and Goodman, 2007). Proponents of structured judgement models of assessment suggest actuarial predictions of future (discrete acts of) abuse are problematic due to the ongoing nature of coercive control (see below). Rather, it is suggested risk assessment should prioritise identifying (combinations of) abusive behaviours and risk factors to intervene and manage the threat of harm. The outputs of both models are subject to wider concerns around what is empirically measurable and what remains inestimable in relation to risk, and the issue of the extent to which ‘human agency’ influences the process both in relation to agencies’ processes for arriving at risk decisions and subjects’ propensity for ‘doing otherwise’ in relation to engaging both with risk identification and management (Walklate and Mythen, 2011:104). The ultimate purpose of risk assessment is, then, contested, with the most cynical view suggesting the process may benefit agencies most by making them appear accountable (Walklate and Mythen, 2011).

Coercive control is another concept that has defied agreed definition and measurement (Hamberger et al., 2017). Building on a long tradition of feminist-activist enquiry, Stark (2007) presents gender-based violence as a human rights issue suggesting the most common manifestation of abuse requiring agency intervention is a course of coercive and controlling conduct comprising multiple abusive behaviours and tactics. Significantly, in the context of risk assessment, Stark (2007) illustrates that in many contexts physical violence may not be the most salient form of abuse and that sometimes the threat of violence alone is enough to reinforce non-physically abusive coercion and control. Barlow and Walklate (2021) suggest the ongoing and multi-faceted nature of coercive control is problematic for risk assessment that is typically one point in time and filtered through an operational lens of frontline policing which focuses primarily on discrete physical assaults while ignoring wider (gendered) contexts and victims’ own perceptions of risk.

## The development of risk assessment for domestic abuse in England and Wales

In 2005, in recognition that risk assessment tools and management models were not being developed and implemented consistently, the Association of Chief Police Officers (ACPO)^1^ published guidance on identifying, assessing and managing risk in cases of DA. In 2007, a proposal from within the police service to develop a common risk model for DA was accepted by ACPO. An expert panel was formed, chaired by the ACPO lead officer for DA. The panel sought to develop a risk model drawing primarily on two existing risk tools: SPECSS + (Metropolitan Police Service), and the ‘FSU9’ checklist (South Wales Police); the latter formed the basis of the CAADA^2^ Risk Identification Checklist used by partner agencies. Both the South Wales checklist (see Robinson, 2006) and SPECSS + (see Richards, 2006) were based initially on analysis of cases of domestic homicide. The developers of both tools considered existing international literature and expertise on risk assessment, as well as seeking the views of survivors of DA (see Richards, 2006; Robinson, 2006), but there is no published record of the precise (iterative) development process for these tools. SPECSS + was subject to a process evaluation (Humphreys et al., 2005) which noted, among other things, that the model was implemented in different ways across pilot sites, reluctance among first response officers to complete the assessment for cases they regarded as low-risk, and concern from specialist officers about increased workload.

In 2009, ACPO endorsed the DASH as a standard model for identifying, assessing and managing risk. The DASH was developed by the expert panel, which comprised police practitioners, academics and representatives from CAADA, over an 18-month period (Robinson, 2010). DASH was intended to ensure a consistent approach to risk between forces, and also that a consistent approach was taken by the police, other statutory agencies and voluntary sector organisations. The DASH assessment consists of 27 questions^3^ to be asked of victims of DA by first responding officers at the scene, or as soon as possible following a call to police. The initial DASH questions are yes/no responses; officers are expected to probe and ask follow-up questions and record freetext information to contextualise the yes/no response. Following completion of the DASH, officers are required to allocate a risk grade of ‘standard’, ‘medium’ or ‘high’.

It is apparent that the question content, wording and ordering of the final iteration of the DASH differs markedly from earlier iterations of both the South Wales checklist (see Howarth et al., 2009) and SPECSS + (see Richards, 2003), and it was not straightforward to achieve consensus in the expert panel as to the final format of DASH.^4^ We have found no published source documenting in any detail the process of development of DASH undertaken by the panel. While it might be possible to say that DASH has a degree of ‘face’ or content validity, it remains unclear how comprehensive or robust were the processes of question design and testing specifically.

## Evaluation of the DASH model

It was also not straightforward to achieve consensus in the expert panel as to the need or otherwise for testing and evaluating the final iteration of DASH.^5^ According to a paper presented to ACPO Chief Constable's Council, DASH was piloted in divisions of four police forces, one specialist DA unit, and with four specialist DA support services (ACPO, 2009a). Though it suggested DASH had been well received by officers, there was no specific evaluation report or reports presented with the summary paper considered by APCO Council, and the authors could not find any published outputs from these pilots. It would appear then that the DASH was endorsed by national policing leads in the absence of peer reviewed evaluation.

Following evaluation of a pilot project concerned with discretionary use of risk assessment (Myhill, 2016), and a highly critical 2014 thematic inspection of police response to DA in England and Wales (Her Majesty's Inspectorate of Constabulary, 2014), the College of Policing worked with academics to review the operation of the DASH risk model in three forces (Robinson et al., 2016). The review found the DASH was not applied consistently and that officers displayed an over-focus on physical assault and injury when making judgements about risk. It concluded there should be an evidence-based approach to risk assessment, and that ‘an understanding of coercive control needs to be embedded within a risk-led approach’ (Robinson et al., 2016:i). This recommendation is supported by Myhill and Hohl's (2019) analysis of DASH data which found coercive control to be the ‘golden thread’ running through identification, assessment and management of risk (see also Myhill et al., 2022, for a discussion of coercive control and policing).

## The present study

The present study provides unique empirical evidence on how officer practice in completing the DASH with victims may give rise to systematic biases that preclude the full and accurate identification of the presence of risks indicative of controlling and coercive behaviour. First, we use multilevel modelling to measure the extent to which responses to DASH questions are explained by factors pertaining to the *officer* completing the DASH interview, rather than by factors pertaining to the *suspected abuse* that the DASH intends to capture through the victim's responses to the questions. A certain amount of measurement error of this kind is to be expected. It seems both natural and unavoidable that certain officer-level variables may influence victims’ ability and readiness to disclose the ‘true’ answer to any given DASH question. For example, officer demeanour when introducing and undertaking the risk assessment and whether the officer tends to give victims time and space to take in and respond to the questions are officer-level variables that may influence a victim's responses. Effects of this nature are well-documented within survey research and termed ‘interviewer bias’ or ‘interviewer effect’. Interviewer effects broadly fall into three categories. First, systematic under-reporting of socially undesirable opinions, behaviours or traits (alcohol abuse, e.g.) due to the presence of the interviewer asking the question and hearing/recording the response. Second, altered reporting because of observable interviewer characteristics that may seem related to the interview question (such as interviewer gender, age or ethnicity). Third, altered reporting as a consequence of interviewer experience or style (Groves et al., 2009). Most studies find this effect to be small, explaining 1.5% to 3.2% of the variance in survey responses (Sturgis et al., 2021). However, some studies have found significantly larger effects. For example, Schuman and Converse (1971) observed that the percentage of respondents agreeing with the statement ‘most White people can trusted’ was five times higher when the interviewer was White compared to when the interviewer was Black.

Returning to risk assessments for DA, if officer-effects shape victims’ responses in a substantial and systematic way it would significantly reduce the accuracy and reliability of the DASH as a tool for identifying risk. Further, if the size of the officer-effect varies systematically between DASH questions in such a way that particular types of abusive behaviours or risk factors are less likely to be identified (correctly, or at all) it would result in an incomplete assessment of risk to victims of those types of behaviours, and a less appropriate safeguarding response. For example, existing research suggests officers tend to over-focus on physical violence at the present incident and do not always consider underlying patterns of ongoing abuse, particularly of coercive control (Barlow and Walklate, 2021; Robinson et al., 2018).

The present study seeks to address three questions. First, to what extent do ‘officer-effects’ influence the probability of risk factors being disclosed and recorded as part of the DASH interview? Second, does the presence and size of such effects vary between DASH questions? Third, can the quantitative findings be corroborated and explained through interviews with and observations of frontline officers completing DASH assessments? We find evidence of an officer effect, pertaining to some questions more than others, and this effect is evident in the way officers undertake and speak about the process of risk assessment. Although focused on the DASH specifically, the study has implications for similar interactions between frontline practitioners and victims of crime in any jurisdiction in which (risk) data is collected and recorded, and for research that employs data ‘constructed’ during these types of encounters.

## Research methods

### Quantitative data and method of analysis

The dataset consists of all 118,512 DASH risk assessments completed between the 1^st^ of March 2018 and 30^th^ of April 2021 by a total of 3079 officers in one English police force. The police force is of medium-large size, with average levels of DA compared to other English forces (Her Majesty's Inspectorate of Constabulary Fire and Rescue Services, 2021). The census sampling method means the data are representative of all DASH assessments completed within the three-year study period. The analysis is based on the yes/no responses to the 27 DASH questions, and a unique person identification number identifying the officer completing the DASH assessment.

99.62% of the 3076 officers in the sample completed more than one DASH. As a result, the dataset has a hierarchical structure with DASH assessments completed by the same officer ‘nested’ or ‘clustered’ in officers. This nesting of DASH assessments in officers is the key feature of interest. A multilevel model allows for quantifying the proportion of variance in responses to a particular DASH question that is shared between DASH assessments completed by the same officer but not shared between DASH assessments completed by different officers; in other words, the extent to which DASH responses are similar because they are recorded by the same officer and as such explained by officer-level characteristics rather than explained by the suspected abuse the DASH intends to capture.

We specify a logistic random-intercept model where i denotes individual DASH assessments (level 1) and j denotes officers (level 2) for DASH item y_1_

$$\mathrm{logit}\{Pr(y_{1ij}=1|u_{j})\}=\beta_{0}+u_{j}$$

with *u*_j_∼N(0,σ*_u_*^2^) and *u* independent across officers j. Pr(y_1ij_=1|*u*_j_) is the probability of DASH item y_1_ in DASH i completed by officer j taking the value 1 (i.e., a ‘yes’ response is recorded); β_0_ is the log-odds that y = 1 when *u *= 0. *u*_j_ is the effect of officer j on the log odds that y = 1. We repeat this analysis for all 27 DASH questions y_1_ to y_27_. We then calculate the intra-class correlation coefficient (ICC) for items y_1_ to y_27_ to estimate the between-officer variance relative to the within-officer variance. The interpretation of the ICC in this context is as a measure of the extent to which the police recorded response to a particular DASH item is explained by the sum of officer-level (unobserved) officer-related variables (level 2) – for example, officer characteristics, demeanour, attitude, knowledge – rather than by the suspected abuse (level 1) the item intends to capture. As a rule of thumb, an ICC larger than 0.05 is considered a small-to-medium effect, and anything with an ICC of 0.1 or above is considered large (LeBreton and Senter, 2008).

Grogger et al. (2021) found that over 40% of DA incidents attended by police involved the same victim and offender. As a result, we expect there to be clustering of DASH assessments in victims (victims having completed more than one DASH over the three-year study period). The dataset provided to us did not contain a victim person identifier that could be used to control for this issue; implications are discussed in the limitations section.

The 3076 officers in the dataset completed an average of 38.5 DASH assessments each, with a minimum of 1 and a maximum of 974 DASH assessments completed per officer. There were a total of 21 outliers of officers apparently completing more than 380 DASH assessments over the three-year study period. To test the sensitivity of the analysis to these outliers and check the robustness of findings we re-ran the logistic random intercept regression excluding these outliers. Excluding outliers did not change results beyond the third decimal digit from the decimal point, and the results presented in the findings section are those of the full sample.

### Qualitative data

The qualitative data that informs this paper is drawn from three projects undertaken over the past decade in different police force areas. The first author led these projects; the third author was a co-investigator and undertook fieldwork for two. Data for Project 1 was collected between 2012 and 2014 in a medium-sized force in the south of England and comprised approximately 110 h of field observations with first response officers and 32 in-depth interviews (see Myhill, 2016; Myhill and Johnson, 2016). Data for Project 2 was collected from late 2015 to 2016 in one Welsh and two English forces and comprised around 120 h of field observations with first response officers alongside a total of 35 in-depth interviews with police officers and staff involved in all aspects of the risk assessment process (see Robinson et al., 2016). Data for Project 3 was collected in two English forces between 2019 and 2020 and involved approximately 350 h of observations with first response officers and DA investigators incorporating ethnographic interviews (see Myhill et al., 2022). In total, the fieldwork across the three projects covered six forces including larger and smaller forces, urban and rural areas, and a range of geographical locations.

Field observations across the three projects totalled around 580 h. Researchers accompanied officers for the duration of their shift, taking the role of ‘observer as participant’ (McNaughton Nicholls et al., 2014:247). Researchers took scratch notes or ‘jottings’ (Emerson et al., 1995) while on shift; field observations and ethnographic interviews were written up subsequently as detailed fieldnotes comprising description, observer comments and subjective reflections (McNaughton Nicholls et al., 2014:260), typically within 48 h of the observed shift. In-depth interviews were recorded and professionally transcribed.

Data from in-depth interviews was analysed using the ‘framework’ method (Ritchie and Lewis, 2012). A coding framework was developed informed both by prior research and inductively through the data (see Boyatzis, 1998). Interview transcripts were coded, and thematic charts created based on the coded data. Anonymised fieldnotes from the observations were exported into the qualitative software analysis programme NVivo. The data were coded iteratively using the principles of grounded theory (see Charmaz, 2014). Coding involved the identification of initial themes which were subsequently examined and synthesised across the research team to develop an agreed coding system. Further themes and codes were identified and refined as analysis progressed.

The force that provided quantitative data was different to any of the forces involved in the qualitative fieldwork.

## Quantitative findings

Table 1 displays the results of the analysis. To begin with basic descriptive statistics, the mean sum score is 4.4 recorded ‘yes’ responses per completed DASH. Table 1 shows considerable variability in the prevalence of risk factors recorded in the DASH. The abuser having problems with drugs, alcohol or mental health is recorded as present on 55% of forms, recent separations or attempts to separate in 47%, and the abuser having previously been in trouble with police in 38%. A third of DASH assessments show the victim disclosing they are very frightened (34%), and about a fifth to a quarter indicate the victim disclosed the abuse was getting worse (22%), the abuse was happening more often (24%), or being afraid of further injury or violence (24%). Rarely disclosed and recorded are the abuser ever having threatened to hurt or kill the children (1%), having hurt the children (1%), having mistreated an animal or family pet (3%), sexual coercion and abuse (4%), or the abuser using a weapon to hurt the victim (7%).

**Table 1.** DASH item response and officer intra-class correlation (ICC).

Item	Question	% Yes	ICC
1	Has the current incident resulted in injury?	13.9%	0.07
9	Are you currently pregnant or have you recently had a baby in the past 18 months?	8.2%	0.08
7	Is there conflict over child contact?	14.7%	0.08
21	Has the abuser had problems in the last year with drugs (prescription or other), alcohol or mental health leading to problems in leading a normal life?	54.8%	0.11
2	Are you very frightened?	34.0%	0.13
6	Have you separated or tried to separate from your abuser within the past year?	46.8%	0.13
3	What are you afraid of? Is it further injury or violence?	24.2%	0.13
8	Does the abuser constantly text, call, contact, follow, stalk or harass you?	14.1%	0.15
23	Has the abuser ever breached bail/an injunction and/or any agreement for when they can see you and/or the children?	10.0%	0.16
12	Does the abuser try to control everything you do and/or are they excessively jealous?	20.1%	0.17
26	Has the abuser ever hurt the children/dependents?	1.4%	0.18
27	Has the abuser ever threatened to hurt or kill the children/dependents?	1.0%	0.18
25	Are there any children, step-children who aren't the abuser's that live in the household? Or are there other dependents in the household (i.e., older relative)?	19.5%	0.19
15	Has the abuser ever attempted to strangle/choke/suffocate/drown you?	8.8%	0.20
14	Has the abuser ever threatened to kill you or someone else and you believed them?	8.3%	0.21
17	Is there any other person that has threatened you or that you are afraid of?	4.7%	0.21
13	Has the abuser ever used weapons or objects to hurt you?	6.7%	0.22
20	Are there any financial issues? For example, are you dependent on the abuser for money/have they recently lost their job/other financial issues?	11.1%	0.22
4	Do you feel isolated from family and friends, that is, does the abuser try to stop you from seeing friends/family/Dr or others?	9.5%	0.22
10	Is the abuse happening more often?	24.2%	0.23
11	Is the abuse getting worse?	22.4%	0.23
5	Are you feeling depressed or having suicidal thoughts?	14.3%	0.24
19	Has the abuser ever mistreated an animal or the family pet?	2.8%	0.24
24	Do you know if the abuser has ever been in trouble with the police or has a criminal history?	38.4%	0.27
22	Has the abuser ever threatened or attempted suicide?	14.8%	0.28
16	Does the abuser say or do anything of a sexual nature that make you feel bad, or that physically hurt you or someone else?	4.3%	0.29
18	Do you know if the abuser has hurt anyone else?	11.0%	0.32
	**Mean DASH sum score**	**4**.**4**	**0**.**17**

The ICC column in Table 1 shows the extent to which the probability of a ‘yes’ response being recorded for a particular DASH question is explained by which officer is completing the DASH, rather than the presence of this particular factor in the suspected abuse the item intends to capture. ICC is not significantly correlated with the percentage of ‘yes’ responses to the particular item (*r* = −0.31, *p* = 0.108). Recall Sturgis et al. (2021) found ICCs between 0.015 and 0.032 in their review of survey interviewer effects. In contrast, all 27 DASH items have an ICC greater than 0.05, and 22 out of the 27 have an ICC greater than 0.10. The ICC for the DASH sum score is 0.17, indicating that 17% of the variance in this score is explained by officer-level characteristics, rather than by characteristics of the suspected abuse.

The items with the lowest ICC are the current incident having resulted in physical injury (0.07), the victim being currently pregnant or recently having a baby (0.08), and conflict over child contact (0.08). They have in common a focus on the present incident or circumstances, and do not require disclosure of specific acts of severe physical violence, or directly named forms of coercion or control. These items are least affected by error arising from the measurable impact of officer characteristics on the probability of a ‘yes’ response being recorded; in other words, for these items, the specific officer completing the DASH matters least.

Inter-officer variability is greater and officer characteristics more salient for many of the items Myhill and Hohl (2019) showed form part of a cluster of indicators of controlling and coercive behaviour and ongoing patterns of abuse, as well as specific items officers may find difficult to ask and to which victims may be reluctant to respond. The two items with the highest ICC are the victim disclosing the abuser having hurt anyone else (0.32), which requires the victim to ‘tell’ on the perpetrator, and sexual coercion and abuse (0.29) which tends to be underreported in most contexts. These items are followed by the abuser threatening or attempting suicide (0.28), and the abuser having previously been trouble with police (0.27). For these items, a substantial 32% to 27% of the probability of an affirmative response being recorded is explained by officer characteristics. Further items with a high ICC include the disclosure of sub-lethal violence (strangulation or choking), threats to kill, the use of weapons to hurt the victim, feeling isolated from family and friends, the victim reporting an escalation in frequency or severity of the abuse, the victim disclosing feeling depressed or suicidal, and the abuser being controlling and/or excessively jealous. The ICC for these items suggests that the DASH assessment is less reliable and accurate in identifying the presence and risks associated with controlling and coercive abuse compared to physical injury alone, or ‘circumstantial’ factors (separation, pregnancy, conflict over child contact, the perpetrator's use of drugs and alcohol) that may be present in some cases that don’t involve ongoing patterns of coercion and control, or that may be self-evident or easier to disclose in cases that do.

Also of note are high ICCs for items asked towards the end of the DASH interview. There is a statistically significant, moderate correlation between DASH question order and the ICC (*r* = 0.41, *p* = 0.032). A positive correlation between ICC and question order could result from frequent occurrence of officers stopping the interview before reaching the end of the DASH assessment, officers asking later questions in a manner that leaves victims with less time, space, or comprehension of the question to respond, and/or victims initially engaging with the DASH but disengaging at some pointing during the interview. Any one of these situations would result in no further ‘yes’ responses being recorded to later questions. Unfortunately, the data do not allow distinguishing between the victim having responded and the officer having recorded a ‘no’ response, the victim having refused to answer a particular question, or the question not being asked at all, which would have enabled testing of this hypothesis.

## Qualitative findings

Findings from qualitative research on police response to DA help further illuminate and explain the quantitative findings around the officer effect on risk assessment using the DASH. Relevant and consistent themes emerged across studies undertaken in different forces, across an almost ten-year time period.

### Officer and staff attitudes and understanding

Across all three research projects, it was evident that compliance rates for the DASH risk assessment were not one hundred percent. In part, that might be explained by the wide-ranging official definition of domestic violence and abuse, and the consequent requirement for officers to interpret whether reported incidents involve individuals covered by the definition and/or whether the behaviour reported is inherently abusive. In some instances, it does appear that a DASH risk assessment is not required (see Myhill, 2016). In others, it appears that officers’ understanding of DA, especially non-physical abuse and/or post-separation harassment or stalking, leads them not to undertake a risk assessment when doing so may in fact have identified risk of harm. In the following example, a neighbour reported a man behaving in a threatening manner outside his ex-partner's house. There had been no previous reports to police.

We did not spend long at the scene (10–15 min). [He] and the other officer discussed the incident briefly, concluding that there had been no criminal offences … I asked [him] if this was a domestic incident. He said that it would almost certainly be [classed] as a domestic, in a way that made me think he thought it shouldn’t be. I asked about a DASH and he said one might be required … [contrary to force policy to complete a DASH in all cases] he would not generally consider a DASH to be necessary unless there were criminal offences. (Fieldnotes, first response)

The man's ex-partner, who was not home at the time, was not contacted subsequently to undertake a DASH assessment. A few weeks after this incident, the victim sustained serious injuries jumping from a first-floor window to escape an attack from the perpetrator.

While some officers appear willing not to submit a risk assessment in certain cases, many officers across the three research projects suggested that it is frequently easier to complete a DASH than to argue the case for not completing one. This situation can result in assessments being submitted in cases where the officer has not asked the questions of the victim, or at least not asked them in a structured or complete way.

Interviewer: Do you fill one in at every incident?

Respondent: No … because I’ll go to situations where … it's given the title of domestic incident and … it won’t be a domestic incident and no matter how well you try and explain to control room that it's not a domestic incident … they’ll say … you’ve got to do a DASH.

Interviewer: So you would then have to do one?

Respondent: I’d do a DASH. I won’t call [the victim] back, I will just sit there and do a DASH from the discussion we had. As silly as it sounds, I will then try and make that DASH sound like a low risk just to prove my point that it wasn’t a domestic. (Interview, first response)

Clearly, DASH assessments submitted based on an unstructured initial account and the officer's gut feeling risk misrepresenting the threat of harm to the victim; previous research showing how victims, for a number of valid reasons, frequently minimise the severity of abuse (Kelly, 1999) suggests the most likely outcome is a poor understanding and underestimate of risk.

It is also apparent that some officers (still) hold sceptical attitudes towards (some) victims of DA and the reports of abuse they make. Across the research projects, officers distinguished frequently between ‘genuine’ domestics/victims and what they would most often describe as ‘arguments’. Another prominent theme is officers’ sense that many reports of DA are intended to get back at somebody or gain the victim some kind of advantage.

He told me about a case where [he] had asked the DASH questions and ‘the stuff the woman came out with’ was, in his view, ‘nothing’, they’d just had an argument, but it all still had to be written down because she had said ‘yes’ to the questions. He used this as an example of the ways police responses to domestics could be used as a form of ‘point scoring’. (Fieldnotes, first response)

The other officer … said she thought victims occasionally exaggerate and say ‘yes’ to everything to try and get the perpetrator into greater trouble (‘a woman scorned’). (Fieldnotes, first response)

Even in cases where specific and highly concerning abuse was reported, some officers showed a tendency to downplay the seriousness of the disclosures during the DASH interview.

For the control question, the woman said he was ‘very old fashioned’ and ‘always had to get his way, or there’d be trouble’ so she would ‘do what he wants to keep the peace’, but the officer put this as a ‘no’. When she said ‘yes’ to the strangulation question, he spent a while asking her about what had happened – whether he had ‘just grabbed her’, or if he had ‘squeezed tightly’, and she confirmed the latter as ‘yeah gripping, squeezing sort of thing’, but … the strangulation answer was put down as a ‘no’. In the briefing, he described the behaviour as ‘grabbing her by the throat’. (Fieldnotes, first response)

There were examples of officers suggesting that, as a matter of course, they ‘interpret’ a victim's responses to the questions and record what they believe to be the appropriate answer (whether or not this answer reflects the victim's initial response). This practice is concerning bearing in mind research has shown victims to be good judges of the threat posed to them by their abusers (see Barlow and Walklate, 2021).

The officer said that he doesn’t always go with the victim's initial yes/no response when completing the risk assessment, but rather uses his judgement to interpret what constitutes a ‘definite yes’. There appeared however to be inconsistency in this approach: [He] recorded a ‘yes’ response to the question on threats to kill, while recording in the freetext that the victim said she did not believe the threats … I noticed he had recorded ‘no’ to the question on controlling and jealous behaviour, despite his notes reading the victim saying ‘yes/he keeps tabs on me’. (Fieldnotes, first response)

A further problematic example of officers using judgement or guesswork concerned the recording of responses for questions that had not been asked. We observed numerous examples of officers choosing not to ask certain DASH questions if, for example, they felt they were inappropriate or not relevant to the case in question (see below). In such cases, some officers would record a ‘no’ instead of leaving it blank or checking a ‘not stated’ box (if there was one).

Interviewer: So when you complete the form … [you’ve said you ask only] the most pertinent questions, how do you fill in the other questions? Do you leave them blank, or do you tick no?

Respondent: I think it would probably be a no … If the [questions] have no pertinence I would be filling in no, which … you could probably say was incorrect, because you're almost guessing their answer, but I stick my hand up to that one! (Interview, first response)

This practice is concerning also because it is not clear some officers have the understanding required to know (consistently) whether specific questions are relevant to specific situations. In addition, an (unpublished) research report by the second author suggested that cases where most responses are ‘missing’, or marked as withheld, have more in common with those containing a lot of ‘yes’ responses suggesting minimisation on the part of (high risk) victims and that the distinction between withheld and genuinely negative responses is an operationally useful one. Also observed was a variation on omitting questions whereby officers would ask a question in a leading way which presupposed a victim's response.

The officer used a very leading style of questioning for example, ‘he doesn’t control you, does he?’ (Fieldnotes, first response)

Taken together, these findings suggest attitudes and understanding play a significant role in explaining the officer effect on risk assessment.

### DASH content and design

Though officers’ attitudes to and understanding of DA appeared to influence both their inclination to complete risk assessments and the quality of assessments, the nature of the DASH risk tool itself also appears to play a significant role. While there is general support for the principle of risk assessment (see Robinson et al., 2016), many officers highlighted what they considered to be problems with the DASH model specifically. One issue is the imprecise way in which some of the questions are phrased.

The officer suggested many of the questions on the DASH risk assessment were too loosely worded … He said a lot of people pay attention to the question on fear, but “f*** me, that's subjective”. He also gave the example of somebody saying they felt a bit depressed as opposed to being clinically diagnosed with depression. (Fieldnotes, Domestic Abuse Unit)

Several DASH questions have a main clause followed by one or more follow-up questions or prompts. In addition, some questions conflate more than one concept, such as feeling isolated and the perpetrator's controlling behaviour (item 4), and jealously and the perpetrator's controlling behaviour (item 12). The imprecise nature of some DASH questions results in some officers asking only specific parts of questions and/or summarising or paraphrasing questions in their own words.

The officer softened some of the [DASH] questions/phrased them colloquially (but also incorrectly/completely changing their meaning) … discussing the financial abuse question [he] asked ‘Is he having money problems, like is he skint?’ (Fieldnotes, first response)

The content of the DASH assessment is also a concern for some first response officers. The evidence-base for the DASH, and similar risk assessments, draws largely on research conducted on cases of men's coercive and controlling abuse in heterosexual relationships. The DASH is applied, however, not only to cases involving family members (such as adolescent to parent violence, or fights between siblings), but also to cases involving intimate partners that officers regard (sometimes erroneously) as low-level disputes or arguments. It is clear many officers regard some of the DASH questions to be irrelevant or inappropriate to some of the incidents to which they respond.

[He] cited frustration that [the DASH] presumes every domestic you go to is abuse rather than an argument … He commented how he feels like a ‘complete idiot’ when attending a ‘normal argument’ and being expected to ask the ‘sexual questions’, or the completely ‘out there, mental questions about pets’. This conversation suggested to me he feels these are so inappropriate that he does not ask them in these circumstances. (Fieldnotes, first response)

In some cases, it appeared officers used their professional judgement sensibly in not asking certain questions. In the following example, the DASH interview was with an elderly lady with early-stage dementia. Her son and part-time carer appeared to be having a psychotic episode.

Not all questions asked, and I did not disagree with that approach on this occasion … Officer asked the woman if her son had ever been violent towards her and used that as a proxy for for example, choking, weapons questions. He said outright that it was not appropriate to ask the sexual question and stated it also on the form. (Fieldnotes, first response)

This wide application of the DASH can result then in officers ‘improvising’ and altering the risk assessment to try to fit the situation they are faced with (or that they perceive they are faced with). This approach is especially evident in officers who try to build rapport by weaving the questions into the conversation, as opposed to introducing the DASH and asking them more formally. This less or more formal approach appeared in some cases to be dictated by how serious the officers perceived the case to be.

Respondent: I did go through it with her but very, very … I mean I've been a police officer for five years so you kind of get to know the risk assessment … I think the most pertinent questions … you can pick out in your head and can go through them quite happily with the person that you're dealing with … I also do have copies of the form that if it was a serious, not a serious because it's probably the wrong word to use, if it was a kind of top-end domestic I'd probably be filling it in there and then. (Interview, first response)

It is worth repeating that while in some cases officers need to use legitimately their professional judgement when completing the DASH assessment (such as not asking the question about pregnancy in relation to a fight between two brothers), the practice of asking questions selectively is more concerning in relation to cases involving intimate partners. It is well documented that coercive control can be a subtle and highly personalised form of abuse (Stark, 2007), and that victims may seek to minimise the abuse they are suffering (Kelly, 1999). It is a risky practice therefore for officers to assume, based on their general reading of an ‘incident’, that there is not ongoing abuse underlying what might be presented by both perpetrator and victim as an ‘argument’. Put simply, ‘you don’t know what you don’t know’.

A further issue concerns the recording of information, even in instances where the full question set has been asked of the victim. Where officers conduct the DASH interview either from memory or using an aide memoire and make notes in their pocket notebook for transcription to force systems later in the shift (more common by far in our observations than direct data entry via a mobile device), there is clearly greater scope for incomplete or inaccurate capture of responses. We observed numerous examples where the final transcription of the DASH data did not match our recollection of what was disclosed by the victim.

I wasn’t sure about the recall of some of the answers and felt that possibly isolation and certainly controlling behaviour should have been ticked. (Fieldnotes, first response)

This detail was also not included in the transcription of the DASH data. [He] recorded ‘no’ responses to the questions on injury, stalking, and control/jealousy. Technically, that was how the victim answered those specific questions, but disclosures at other points in the risk assessment suggested otherwise … Crucially, he did not mention in the freetext the perpetrator's stalking behaviours/jealousy. (Fieldnotes, first response)

It is likely that the questions on stalking and harassment and controlling behaviour are more open to interpretation – by officers and victims – than the more directly observable questions, such as physical injury. In the above example, the victim responded ‘no’ to the question on stalking and harassment because it is worded as ‘constantly’ engages in stalking-type behaviours and she said the abuse from her ex-partner was not constant. She disclosed, however, at least two separate examples of worrying stalking-type behaviour (including her ex-partner waiting outside the house of her male friend) – enough to satisfy the requirement of ‘two or more occasions’ for a course of conduct offence in England and Wales.

## Discussion

Our quantitative and qualitative findings evidence that the process of undertaking a DASH assessment is highly variable, depending both on the officer conducting the interview and the nature of the report. Specifically, officer-related characteristics influence victims’ responses to the DASH questions and/or the recording of responses to those questions, and whether in fact an assessment is conducted at all. Significantly, these officer characteristics appear to affect adversely collection and recording of data relating to some risk factors more than others. The officer effect has a much larger bearing on how victims respond to questions on, and how officers record information about, ongoing controlling and coercive behaviour than for questions asking about physical injuries sustained during the most recent incident, or more circumstantial factors (pregnancy, separation, the abuser's use of drugs and alcohol). Qualitative work with first response officers suggests such officer-related characteristics include understanding of DA and coercive control, diligence and professional curiosity, and interpersonal skills relating to building rapport with victims and asking the questions in a way that encourages disclosure. Our findings also suggest that the structure of the DASH itself compounds the officer effect and contributes to the variability in primary assessments of risk. The multi-site nature of our fieldwork increases the likelihood our findings are transferrable to policing in England and Wales; our findings are also consistent with Grant and Rowe (2011) who found variability in knowledge and understanding among police officers in New Zealand, and similar inconsistency in the implementation of primary risk assessments.

We should emphasise again that, across multiple projects, we have observed conscientious officers complete thorough and detailed risk assessments. We concentrated here deliberately on observed shortcomings of the process as it is they that pose obvious problems for the assessment and management of risk. To that end, our findings have a number of implications for the design and implementation of risk tools, safeguarding and referrals that flow from the risk assessment process, and for research that seeks increasingly to use police data, including that collected via the DASH, for the purpose of creating predictive models.

In terms of the design and implementation of frontline risk tools, our findings suggest some practitioners find the imprecise nature of some DASH questions difficult. The DASH guidance acknowledges ‘some questions may appear to overlap’, but with the intention to ‘encourage maximum opportunity for disclosure from victims’ (ACPO, 2009b:2). Our data suggests, however, that the wide-ranging nature of some questions and subsequent prompts is perhaps best suited to administration by specialist support workers who have the time to probe fully victims’ responses and record comprehensive freetext. The structure of some DASH questions and follow-up prompts, especially those in which two concepts are mentioned in the same question, lends itself to paraphrasing by police officers. Good question design seeks to minimise measurement error, including measurement error of this kind. Items with non-negligible ICCs might need to be reviewed to reduce ambiguity and consequent risk of officers altering the question or omitting it altogether. Reducing measurement error in this way would provide greater confidence in responses to yes/no questions where officers have neglected to provide additional freetext explanation.

In relation to safeguarding, it is difficult, currently, based on the variability in officer practice observed, to be confident that any specific risk factor measured by the DASH is present, or more crucially not present, in any given case. An officer effect creates a ‘lottery’ for victims in terms of how their report to police will be assessed and is especially concerning when considering how DASH data is used to initiate formal safeguarding procedures. For non-police users of DASH, SafeLives advocate a threshold of 14 ‘yes’ responses for referral to a Multi-agency risk assessment conference (MARAC), and a ‘mechanistic’ summation of risk factors was also observed in the evaluation of SPECSS + (Humphreys et al., 2005:9). We have found no (published) empirical justification for an actuarial threshold for DASH. The 14 tick threshold has been adopted by some police forces, despite guidance to forces stating DASH does not provide a measure of risk using ‘cut-off scores’ (ACPO, 2009c) and despite the police version of DASH containing three additional questions relating to children which will not be applicable to some victims. In some cases, this threshold appears to stifle the professional judgement that DASH was intended to inform; we witnessed officers referring to cases as for example ‘high-medium’ and appearing less comfortable allocating a risk grade if a threshold number of ‘yes’ responses was not reached. Conversely, we also witnessed examples of risk-aversion and ‘gaming’ of the system to ensure a case received a specific response.

Even though they thought the case was low risk, they said they would put the risk down as a medium because they felt that ‘mediums’ at least got some level of intervention – and they wanted the family to receive bereavement support – while ‘standards’ were basically closed ‘as is’ … only high risk victims were funded to receive safeguarding such as panic alarms … Another victim (graded as ‘medium risk’ but who the officer thought should have been graded ‘high’) had said she was keen to engage with police and receive this support because she was so scared for her safety but was turned down because she couldn’t be funded as a ‘non-high risk’ victim. (Fieldnotes, first response)

As the above example suggests, in many instances, the level of support provided to a victim, be it police safeguarding and intervention or referral to specialist support services, is dictated by the risk grade generated through the DASH. If, as our data suggest, reliable identification of risk through the DASH is biased, with identification of controlling and coercive abuse not involving physical assault during the most recent incident being subject to particularly substantial officer-induced measurement error, it would appear dangerous and unethical to allocate potentially life-saving interventions based solely on the DASH score. The officer bias against reliable identification and recording of controlling and coercive behaviour is especially concerning considering the growing body of evidence associating coercive control with intimate partner homicide (Dobash and Dobash, 2015; Johnson et al., 2019; Monckton Smith, 2020) and suicide (Bates et al., 2021). Simply counting risk factors is not adequate; professional judgement and awareness of context and the interaction between risk factors is required.

A further concern is that inconsistent and inaccurate recording of risk data does not just affect a victim at a single point in time. Although guidance on risk management within the DASH model suggests assessments are ‘kept under review’, in our experience most cases tend to be reassessed only when a further call is made to police. In other words, once a victim is tagged at a specific level of risk, that assessment may follow them for weeks or months and dictate subsequent intervention or follow-up. In addition, robust profiles of offenders and offending behaviour will also be affected by inadequate data, and the underrecording of coercive control in particular, impacting for example applications made by different victims under the Domestic Violence Disclosure Scheme for which thresholds for disclosure have been shown to differ between forces (Hadjimatheou and Grace, 2021).

Systematic bias in the collection and recording of DASH data also has significant implications for secondary analysis of that data. There has been an increase in research using large datasets and machine learning to develop predictive models to forecast future DA victimisation (see e.g. Grogger et al. 2021). As well as the DASH data itself, machine learning models have employed criminal histories and crime harm severity scores, both of which are derived from police recorded crime data. Crime codes themselves are based on the primary investigation by attending officers, part of which constitutes the DASH assessment. If, as our data shows, the DASH process fails to identify and record controlling and coercive behaviour at the rate it is present, and officers systematically under-record coercive control relative to physical assaults (Barlow et al., 2020), this type of analysis risks creating the type of negative ‘feedback loop’ recognised in the operation of algorithms more generally, and predictive policing specifically (see Fry, 2018:186). While there is merit in predicting cases with a higher likelihood of future physical violence, models trained on biased input data will inevitably struggle to predict wider harm, and especially cases involving little physical violence but high and harmful levels of coercion and control.

Following the review of DASH (Robinson et al., 2016), the College of Policing, in consultation with police practitioners, victim-survivors, specialist support services and academics, designed and piloted an alternative primary risk assessment for frontline police. The Domestic Abuse Risk Assessment (DARA) sought to address some of the perceived shortcomings of DASH in a frontline context by simplifying questions and introducing scaled responses for questions in order to reduce the reliance on freetext. Rather than yes/no responses, victims are asked ‘how often’ they experience specific abusive behaviours. Piloting in three forces suggested officers made more appropriate assessments of risk when using the DARA, and documented controlling and coercive behaviour more frequently than when using the DASH (Wire and Myhill, 2018). Yet better identification of coercive control did not always translate to an appropriate assessment of risk, suggesting that improvements to the risk tool itself can only take you so far. Indeed, senior practitioners expressed, prior to the development of DASH, concern that the introduction of a primary risk assessment may be problematic without adequate understanding on the part of those tasked to deploy it:

‘Simplification of the complex issue of risk in the context of policing domestic violence by assigning categories of risk (e.g., ‘high’, ‘medium’, ‘standard’) may be misleading and potentially unsafe, particularly if it is not accompanied by detailed supporting training, information about the nature of the risk, guidance as to how to categorise risk and the consequences … of different categories of risk’ (ACPO, 2005: 8).

While the DARA was designed to preclude simple summation of risk factors, officers ‘grading’ of risk may still be inconsistent if (some) officers maintain a ‘tunnel vision’ focus on physical assault (Barlow and Walklate, 2021:897), while ignoring the salience of non-physical coercion. Officers having a good understanding of DA and coercive control in particular is crucial to the quality of first response, including primary assessment of risk of harm. There is some hope that training adopted at the time of writing by around half of forces in England and Wales may help in this respect (see Brennan et al., 2021).

## Limitations

The quantitative study is based on a large, representative dataset of DASH assessments and officers. Our multi-level model accounts for the nesting of DASH assessments in officers, but in the absence of useable victim and suspect person identifiers our model does not account for the cross-classification of assessments in officers completing the DASH, victims answering the DASH questions, and victim-suspect dyads, or potential interaction effects between the three. If ICCs for victims and victim-suspect dyads are large, it can lead to less accurate standard error estimates and significance tests and underestimation of higher-level (in this case officer-level) variance (Chen, 2012). Consequently, the results presented here are conservative estimates of the magnitude of the officer effect on whether or not a risk factor is recorded on the DASH.

We did not explore the potential effect of officer sex on conducting risk assessments; this might be an avenue for future research.

## Concluding remarks

The process for designing the DASH involved drawing on multiple existing risk tools, including one developed in part for use by specialist support services. The final iteration of DASH, with its multi-pronged questions and requirement for recording contextual freetext information to describe yes/no responses, appears more suited to specialist support workers and less suited to some interactions between police officers and victims at a point of crisis. This situation results in significant variability in the way the DASH is administered, with implications both for safeguarding victims and secondary analysis of risk assessment data. While the (re)design of risk tools may optimise their validity and accuracy, it is equally crucial practitioners have an understanding of DA, as well as the interpersonal skills to build rapport with victims.

The wider question of the ultimate value of risk assessment remains an open one. Though we have identified considerable issues with how risk assessment operates currently, we don’t believe a risk-based approach is ‘inherently flawed’ (Barlow and Walklate, 2021:898). We agree with Mythen (2014:68) that:

‘Rather than typecasting the concept of risk as an *enfant terrible* … we are perhaps better served reminding ourselves of the value of a critical but open approach. Rather than avowing that modes of risk regulation are an inherently good or bad thing in and of themselves, we should begin by looking at the context of their introduction and consider both the potential benefits and possible drawbacks’.

DASH was introduced to prompt first response officers to engage with victims of DA and ask the types of questions research suggested would identify (potentially) harmful scenarios. We have offered ways in which that process may be improved. In addition, there is no reason to think it is inherently impossible for police and partner agencies to understand DA as a process and to situate risks identified in a primary assessment in a wider (gendered) context. We think on balance a primary risk assessment is beneficial, and caution against ‘throwing the baby out with the bathwater’.

## Notes

1. Now the National Police Chief's Council (NPCC).

2. Coordinated Action Against Domestic Abuse, now known as SafeLives.

3. Although broadly the same, the police DASH has three additional questions (covering abuse to children) to the 24 question DASH used by partner agencies and sponsored by SafeLives.

4. Personal correspondence, first author and members of the expert panel.

5. Personal correspondence, first author and members of the expert panel.
